# Retrospective analysis of illness and liver abscesses in low-risk, crossbred beef cattle

**DOI:** 10.3389/fvets.2026.1875341

**Published:** 2026-07-20

**Authors:** Heather L. Bradford, Larry A. Kuehn, Brittney N. Keel, James E. Wells, Amanda K. Lindholm-Perry

**Affiliations:** U.S. Meat Animal Research Center, Agricultural Research Service, U.S. Department of Agriculture, Clay Center, NE, United States

**Keywords:** antibiotic, beef cattle, liver abscess, oxytetracycline, tulathromycin

## Abstract

**Background:**

Liver abscesses are a major economic concern in beef production, yet the relationship between therapeutic health events and liver abscess development remains poorly understood. This study evaluates how health diagnoses, therapeutic treatments, and the timing of these events across the production cycle relate to liver abscess prevalence in a large population of low-risk, crossbred beef cattle.

**Methods:**

Binary liver abscess phenotypes and lifetime treatment records were analyzed for 9,527 animals using generalized linear mixed models. Health events were summarized by diagnosis, treatment type, and antibiotic class during preweaning, feedlot, and lifetime phases.

**Results:**

Although overall therapeutic antibiotic treatment was not associated with liver abscess development (*p* > 0.10), specific antibiotic classes showed sex-dependent relationships. Steers treated with tulathromycin preweaning or at any time had a reduced risk of liver abscesses compared to untreated steers (*p* = 0.02). In contrast, no effect was detected in heifers. A sex-by-oxytetracycline interaction was also identified (*p* = 0.01) but with no significant pairwise contrasts (*p* > 0.10). Heifers diagnosed with infections, primarily infectious bovine keratoconjunctivitis, were less likely to have liver abscesses than heifers without infections (*p* = 0.03).

**Conclusion:**

These findings indicate that early-life health events, particularly preweaning antibiotic treatments, may have lasting effects on liver abscess susceptibility based on this observational study. Experimental research is needed to validate these results. The research highlights the need for mechanistic studies to clarify biological pathways and support the development of non-antimicrobial prevention strategies.

## 1 Introduction

Liver abscesses cost the beef industry millions annually because of condemned livers and reduced carcass value (1, 2). The prevalence ranges from 10 to 50% within cattle groups (1, 3, 4). During the finishing phase, cattle are fed high-concentrate diets, which can lower ruminal pH and induce acidosis and rumenitis. The most cited etiology is that rumenitis allows bacterial translocation, primarily *Fusobacterium necrophorum*, to the liver, where abscesses form (1, 4–6). Steers have more liver abscesses than heifers (3, 5), making sex differences an important consideration for liver abscess development.

Preventative strategies focus on dietary modifications, such as increasing roughage, increasing particle size, and extending the transition period (4, 5). Tylosin phosphate is the most common and consistently effective dietary additive to reduce liver abscesses when fed throughout the finishing period (4, 7). This antibiotic is a macrolide with effectiveness against Gram-positive and some Gram-negative bacteria. Regulatory changes under the Veterinary Feed Directive now require veterinary oversight (8), prompting interest in alternatives.

Although direct-fed prophylactic antibiotic use to prevent liver abscesses has been well studied, limited research has examined the relationship between therapeutic antibiotic use and liver abscess development. Several antibiotics are labeled to treat foot rot caused by *Fusobacterium necrophorum,* leading to the possibility that treatment for an unrelated diagnosis could also affect liver abscess outcomes. Two studies have evaluated metaphylactic antibiotic treatment at feedlot receiving and found no relationship with liver abscesses (9, 10). These studies evaluated combinations of ceftiofur, florfenicol, tilmicosin, or tulathromycin in steers or heifers with no sex comparison within the study. Liver abscesses and respiratory disease may interact, as cattle with lung consolidation or fibrin tags had more liver abscesses than those without (11). Understanding the relationship between health events, treatment regimens, and liver abscess outcomes could inform novel prevention strategies, improve biological understanding, and refine experimental designs.

The primary objective of this retrospective analysis was to evaluate the association between health diagnoses, corresponding treatments, and the subsequent development of liver abscesses in low-risk beef cattle. A secondary objective was to assess how the timing of health events throughout the production cycle may have influenced the occurrence of liver abscesses at slaughter.

## 2 Materials and methods

Data were obtained from the United States Meat Animal Research Center (USMARC) database. All animal procedures were approved by the USMARC Institutional Animal Care and Use Committee, and cattle were processed at a federally inspected packing plant. This study was a retrospective analysis of liver abscess and health data at USMARC.

### 2.1 Data

#### 2.1.1 Animals

Cattle populations represented the diversity of breeds and crosses used in the US beef industry. Cattle were part of the USMARC Germplasm Evaluation or Selecting Functional Alleles projects in Clay Center, Nebraska. During continuous Germplasm Evaluation, semen was obtained from industry sires from the 18 most common beef breeds, and females were bred using artificial insemination. The Germplasm Evaluation population and breeding structure have been described previously and were designed to estimate heterosis and breed effects (12, 13). The Selecting Functional Alleles population included Angus and three composite breeds developed at USMARC (MARC I: ¼ Braunvieh, ¼ Charolais, ¼ Limousin, 1/8 Angus, 1/8 Hereford; MARC II: ¼ Angus, ¼ Gelbvieh, ¼ Hereford, ¼ Simmental; and MARC III: ¼ Angus, ¼ Hereford,¼ Pinzgauer, ¼ Red Poll). Within each breed, one line underwent genomic selection to remove loss-of-function alleles, and one line was maintained as a control (14).

Across both populations, pedigree-predicted heterosis averaged 87% and ranged from 0 to 100%. Because of the limited representation per breed, breeds were grouped into types. Breed types included British (Angus, Hereford, Red Angus, Red Poll, Shorthorn, South Devon), Continental (Braunvieh, Charolais, Chianina, Gelbvieh, Limousin, Maine Anjou, Pinzgauer, Salers, Simmental, Tarentaise), Brahman, and Other (Bonsmara, Holstein, Romosinuano). Cattle represented a wide range of breed crosses such as F_1_, backcrosses, and matings of different F_1_ crosses (i.e., mating a Hereford × Angus with a Simmental × Charolais, sometimes described as F_1_^2^) and a limited number of purebred cattle (*n* = 213). The average animal was 48% British, 48% Continental, 3% Brahman, and 1% other breed types.

Cattle were born from spring 2010 to spring 2023, with fall calving seasons present from 2017 to 2020. Calves were raised in pasture groups by project type (nested within Germplasm Evaluation and Selecting Functional Alleles) before weaning, and the pasture groups were then commingled in the feedlot. Fall-born calves were vaccinated with a 7-way clostridial vaccine and a modified-live vaccine for bovine rhinotracheitis, bovine virus diarrhea, parainfluenza 3, and bovine respiratory syncytial virus at pre-breeding (~ 4 months before weaning) and weaning. Spring-born calves received the 7-way clostridial vaccine at pre-breeding (~ 3 months before weaning) and pre-conditioning (~ 1 month before weaning), and the modified-live vaccine at pre-breeding, pre-conditioning, and weaning. All calves received an injectable dewormer (moxidectin) for internal parasites before weaning, and the dewormer is not effective against liver flukes.

Contemporary groups were defined by project type, slaughter date, and any experimental treatments, including diet and implant types (*n* = 134). These groups accounted for differences in how cattle were fed and managed within and across years. Contemporary groups were removed if they had fewer than five animals or had no variation in liver abscess phenotypes (*n* = 16 groups, *n* = 126 animals). Cattle were 72% steers, and the average animal was 159 days of age (*sd* = 20 d) at weaning, 462 days of age (*sd* = 30 d) at slaughter, and 606 kg (*sd* = 69 kg) at slaughter. If cattle were not weighed prior to slaughter, a quadratic regression of weight on the last 160 day was used to predict slaughter weight.

#### 2.1.2 Feeding management

In the feedlot, cattle were fed an *ad libitum* ration once daily, and diets differed within and across years. On average, cattle were fed a grower diet for 84 days, a transition diet for 40 days, and a finisher diet for 196 days. Grower diets included a coccidiostat after weaning for cattle born in 2017 or later. For cattle born before 2016, transitions were one or two steps across more days (average 47 days). For cattle born in 2016 or later, transitions consisted of three steps, each lasting 1 to 2 weeks, using a blend of grower and finisher diets (70/30, 50/50, 30/70). Finishing diet compositions are shown in Supplementary Table S1 and had considerable within-year confounding.

Tylosin phosphate and chlortetracycline feeding varied by year, and feeding protocols were categorized accordingly. For tylosin phosphate, cattle were either fed the antibiotic throughout the entire feeding period (*n* = 1,064) or only during the transition period (fed ≤ 15 d; *n* = 178). Each of these groups had a corresponding control group that was not fed tylosin phosphate but was managed over the same timeframes. These groups included the entire feeding-period control (*n* = 517) and the transition-period control (*n* = 1,168). An additional group (*n* = 6,600) included cattle from years when either all or none received tylosin phosphate, making treatment confounded by year effects. For chlortetracycline, cattle were fed for up to 9 days during the grower period, and feeding was classified into four categories:

- Chlortetracycline fed (*n* = 1,246);
- Chlortetracycline control (*n* = 3,187), fed during the same time as chlortetracycline-fed cattle;
- All chlortetracycline (*n* = 1,031), representing the years when all cattle received the drug; and
- No chlortetracycline (*n* = 4,063), representing years when no cattle received the drug.

All and no chlortetracycline groups were confounded by year, like the other groups for tylosin phosphate.

#### 2.1.3 Liver abscess

A trained veterinarian scores livers at slaughter (15). Livers were originally scored with the Elanco liver grading system (16), and scoring transitioned to the presence or absence of liver abscesses because of few animals scored with few, small abscesses. To unify records across time, a binary trait was created to indicate the presence or absence of liver abscesses. In this dataset, 16.8% of cattle had liver abscesses, which was lower than the industry average.

#### 2.1.4 Health management

Cattle health was monitored daily by farm operations staff. Cattle with health concerns were treated by animal research technicians based on clinical signs consistent with standardized case definitions and corresponding treatment protocols. Pneumonia was the most common diagnosis and was characterized by depression, decreased appetite, increased respiration rate, and elevated temperature. The next most common diagnosis was foot rot, diagnosed by sudden acute lameness, swelling between the hooves, hoof crack(s), and/or lesion(s), followed by pinkeye, diagnosed by squinting, watery eyes, light sensitivity, and cloudy eyes. Treatment of non-standard cases involved USMARC veterinary staff. Treated cattle were observed daily until recovery.

This study used individual animal health records for cattle with liver abscess phenotypes. Health records were removed for routine management procedures (castration, dehorning) and experimental sampling (biopsies, blood samples, nasal swabs, and rectal temperatures). Cattle were removed if they were part of a metaphylactic treatment where all animals in a group were treated (*n* = 483), and all metaphylactic treatments occurred before weaning. Hence, this study population included low-risk feedlot cattle with no metaphylactic antibiotic use.

Health records were summarized based on diagnosis, treatment drug type, and antibiotic type. Categories representing less than 1% of animals were grouped together. Health diagnoses included respiratory (pneumonia, diphtheria), infection (cellulitis, ear, eye, scrotal, umbilicus), lameness (abnormal gait, foot rot, fracture, laceration, laminitis), and other (abscess, allergic reaction, birth trauma, bloat, buller, central nervous system, coccidiosis, diarrhea, heart failure, hypo- or hyperthermia, gastrointestinal, laceration, prolapse). Although gastrointestinal conditions were of interest for liver abscesses, too few animals were diagnosed to analyze this category separately.

Therapeutic treatment types were grouped into anti-inflammatory, antibiotic, and other drug types (anesthetic, antihistamine, digestive, electrolyte, fluid, nutritional). To further explore antibiotic use, antibiotics were classified by active ingredient, including ceftiofur, florfenicol, oxytetracycline, tildipirosin, tulathromycin, and others (gamithromycin, penicillin, quinolone, and sulfonamide). The other categories also included tildipirosin during preweaning and florfenicol in the feedlot because of too few treatments during those periods. Antibiotic uses and bacterial targets are shown in Supplementary Table S2.

Treatment records were evaluated during three production phases: preweaning, finishing, and lifetime. During each phase, health records were summarized by whether an animal was treated for each diagnosis, therapeutic treatment type, and therapeutic antibiotic type. This segregation allows for assessing the importance of the timing of health events in relation to liver abscesses at slaughter. Each dataset had records for 9,527 animals. Descriptive statistics by production phase are presented in Table 1.

**Table 1:** Prevalence (%) of health events by sex and production phase in low-risk, crossbred beef cattle.

	Preweaning			Feedlot			Lifetime^a^		
Effect	All	Heifer	Steer	All	Heifer	Steer	All	Heifer	Steer
Diagnosis									
Respiratory	2.7	1.3	3.3	10.6	9.4	11.0	13.0	10.7	14.0
Infection	5.1	4.2	5.4	2.6	2.3	2.7	7.4	6.3	7.8
Lameness	3.2	2.0	3.6	4.5	3.8	4.8	7.5	5.7	8.3
Other diagnosis	1.0	0.4	1.2	1.6	1.4	1.6	2.5	1.8	2.8
Therapeutic treatment type									
Anti-inflammatory	3.0	1.4	3.7	13.0	11.3	13.7	15.6	12.4	16.9
Antibiotic	10.9	7.5	12.3	16.2	14.9	16.7	25.3	21.4	26.9
Other treatment type	0.5	0.1	0.6	1.2	1.1	1.3	1.7	1.3	1.8
Therapeutic antibiotic type									
Ceftiofur	1.5	1.3	1.6	4.9	4.8	4.9	6.3	6.0	6.4
Florfenicol	1.2	0.5	1.4				2.0	0.8	2.4
Oxytetracycline	3.8	3.6	3.9	2.1	1.2	2.4	5.8	4.8	6.2
Tildipirosin				8.0	7.5	8.1	8.0	7.5	8.3
Tulathromycin	5.0	2.3	6.0	1.5	1.5	1.4	6.4	3.8	7.4
Other antibiotic type	1.3	1.1	1.4	1.4	1.0	1.6	1.8	1.7	1.8

Data included 2,710 heifers and 6,817 steers.

a Preweaning and feedlot do not sum to lifetime because animals could be treated during both preweaning and feedlot phases but only count once if they were ever treated in the lifetime data.

### 2.2 Statistical analysis

Binary liver abscesses were analyzed using generalized linear mixed models with a logit link function and fit with the glmer function in the lme4 package 1.1–37 in R 4.4.1 (17, 18). Three datasets corresponding to the preweaning, feedlot, and lifetime phases were analyzed separately to evaluate health effects at different stages of production.

The baseline model was:


$$\begin{array}{c} \text{liver absces}s_{\text{ijkl}}=\beta_{0}+\mathrm{se}x_{i}+\mathrm{age}\;\text{of}\;\mathrm{da}m_{j}+\beta_{1}\text{slaughter}\;\mathrm{ag}e_{k}\\ +\beta_{2}\text{slaughter weigh}t_{k}+\beta_{3}\text{heterosi}s_{k}\\ +\beta_{4}\text{Continental bree}d_{k}+\beta_{5}\text{Brahman bree}d_{k}\\ +\beta_{6}\text{Other bree}d_{k}+\text{grou}p_{l}+e_{\text{ijkl}}. \end{array}$$

Liver abscess was the binary liver abscess phenotype (liver abscess = 1, no liver abscess = 0). The fixed effects were sex (i = steer or heifer) and age of dam (j = 2, 3, 4, 5 to 9, 10+) based on modified Beef Improvement Federation guidelines (19). Fixed covariates included normalized age at slaughter, normalized slaughter weight, heterosis, the proportion of Continental breeds, the proportion of Brahman, and the proportion of other breeds for animal k. The proportion of British breeds was a linear combination of the intercept and all other breed covariates. The group was the contemporary group (l = population type, slaughter date, treatment groups) and was fit as a random effect with constant variance. For weaning analyses, pre-weaning pasture groups were considered in the contemporary group definition, but slaughter contemporary groups provided a better fit for the liver abscess response variable. e was the random residual.

For the feedlot and lifetime analyses, additional fixed effects were included for tylosin phosphate and chlortetracycline feeding. Tylosin contrasts: feeding throughout the finishing period versus its paired control and feeding during the transition period versus its paired control. Chlortetracycline contrasts compared cattle fed chlortetracycline with contemporaneous controls.

For each production phase, three models were used to evaluate health effects:

1. diagnosis models included fixed effects for respiratory, infection, lameness, and other diagnoses;
2. therapeutic treatment type models included fixed effects for anti-inflammatory, antibiotic, and other treatments; and
3. therapeutic antibiotic type models included fixed effects for ceftiofur, florfenicol, oxytetracycline, tildipirosin, tulathromycin, and other antibiotics. Antibiotic types with limited observations during a given phase were grouped into other (tildipirosin preweaning, florfenicol in the feedlot).

In total, these analyses resulted in nine models (three health categories × three production-phase datasets).

Interactions between fixed effects were tested. The final models were selected based on Akaike information criterion (20) and the condition number of the inverse of the coefficient matrix to control for confounding. Significant interactions included sex × infection (preweaning and lifetime), sex × tulathromycin (preweaning and lifetime), and sex × oxytetracycline (lifetime). Final models included predictors with non-empty cells in Tables 2, 3.

**Table 2:** *p*-values for the relationship between health events and liver abscesses during different production phases in low-risk, crossbred beef cattle.

Effect	Preweaning	Feedlot	Lifetime
Diagnosis			
Respiratory	0.71	0.32	0.40
Infection	x^a^	0.25	x
Lameness	0.30	0.40	0.28
Other diagnosis	0.37	0.53	0.52
Sex × infection	**0.05**		**0.02**
Therapeutic treatment type			
Anti-inflammatory	0.52	0.89	0.48
Antibiotic	0.84	0.63	0.97
Other treatment type	0.50	0.99	0.87
Therapeutic antibiotic type			
Ceftiofur	0.80	0.24	0.35
Florfenicol	0.41	-	0.97
Oxytetracycline	0.85	0.71	x
Tildipirosin	-^b^	0.68	0.62
Tulathromycin	x	0.96	x
Other antibiotic type	0.45	0.64	0.36
Sex × oxytetracycline			**0.01**
Sex × tulathromycin	**0.02**		**0.01**

a Involved in an interaction. Main effect results are not shown.

b Not analyzed because too few animals were treated. Bold values are *p*-values less than 0.05.

**Table 3:** *p*-values for animal-specific effects during preweaning, feedlot, and lifetime on liver abscesses in low-risk, crossbred beef cattle.

Effect	Preweaning	Feedlot and lifetime
Age of dam	**< 0.01**	**< 0.01**
Sex	**< 0.01**	**< 0.01**
Slaughter age, scaled	**0.04**	0.15
Slaughter weight, scaled	0.93	0.94
Heterosis	0.89	0.89
Breed	0.14	0.16
Tylosin phosphate feeding	-^a^	**0.03**
Chlortetracycline feeding	-	**< 0.01**

a Not included in the model. Bold values are *p*-values less than 0.05.

Residuals were assessed for overdispersion and heteroscedasticity using the DHARMa package 0.4.7 (21). Significance was defined as a *p*-value of ≤ 0.05, and a trend was 0.05 < *p* ≤ 0.10. For significant interactions, select contrasts were evaluated with a Bonferroni *p*-value adjustment (22) in the emmeans package 1.11.2 (23). Pairwise age of dam contrasts is included in the Tukey adjustment (24).

## 3 Results

### 3.1 Relationship between diagnosis and liver abscesses

*p*-values for the relationship between health events and liver abscesses are shown in Table 2 with all odds ratios included in Supplementary Table S3. Sex and infection interacted during preweaning and lifetime phases (*p* ≤ 0.05). Based on contrast, only heifers had a difference in lifetime data (*p* = 0.03). Heifers with diagnosed infections were 50% less likely to have liver abscesses than heifers without infections in this low-risk population (Table 4). Fewer heifers had infections than steers, and the sexes exhibited different infection patterns based on production phases. Ninety percent of infections were infectious bovine keratoconjunctivitis, or pinkeye. Heifers had more pinkeye preweaning, and steers had more in the feedlot. One-half of pinkeye infections were treated with oxytetracycline, and 21% were treated with tulathromycin. No other diagnoses, including respiratory conditions and lameness, were related to liver abscesses.

**Table 4:** Odds ratios for the significant sex interactions with health traits for liver abscesses in low-risk, crossbred beef cattle.

	Preweaning			Lifetime		
Contrast	Odds ratio [95% CI]	*p*-value	Risk ratio [95% CI]	Odds ratio [95% CI]	*p*-value	Risk ratio [95% CI]
Sex × infection						
Heifers: infection/no infection	0.44 [0.17, 1.13]	0.12	0.48 [0.20, 1.11]	0.44 [0.21, 0.94]	0.03	0.49 [0.24, 0.95]
Steers: infection/no infection	0.98 [0.67, 1.44]	1.00	0.98 [0.71, 1.33]	0.95 [0.69, 1.31]	1.00	0.96 [0.74, 1.23]
Infection: heifer/steer	0.34 [0.13, 0.94]	0.03	0.39 [0.15, 0.95]	0.37 [0.17, 0.84]	0.01	0.43 [0.20, 0.87]
No infection: heifer/steer	0.76 [0.58, 0.99]	0.04	0.80 [0.63, 1.00]	0.80 [0.63, 1.02]	0.10	0.84 [0.68, 1.02]
Sex × tulathromycin						
Heifers: treated/not	1.50 [0.62, 3.60]	1.00	1.40 [0.66, 2.63]	1.44 [0.76, 2.75]	0.63	1.36 [0.78, 2.22]
Steers: treated/not	0.60 [0.39, 0.94]	0.02	0.65 [0.44, 0.95]	0.67 [0.46, 0.98]	0.03	0.73 [0.53, 0.98]
Treated: heifer/steer	1.75 [0.67, 4.60]	0.59	1.60 [0.70, 3.18]	1.08 [0.47, 2.46]	1.00	1.06 [0.52, 1.96]
Not treated: heifer/steer	0.71 [0.54, 0.92]	< 0.01	0.75 [0.59, 0.94]	0.50 [0.32, 0.79]	< 0.01	0.57 [0.38, 0.83]
Sex × oxytetracycline						
Heifer: treated/not				0.50 [0.23, 1.10]	0.11	0.56 [0.27, 1.08]
Steer: treated/not				1.17 [0.82, 1.66]	1.00	1.13 [0.85, 1.47]
Treated: heifer/steer				0.48 [0.20, 1.18]	0.17	0.54 [0.24, 1.14]
Not treated: heifer/steer				1.12 [0.75, 1.69]	1.00	1.10 [0.78, 1.49]

### 3.2 Relationship between therapeutic treatment and liver abscesses

No broad treatment drug types were related to liver abscess prevalence (Table 2). Antibiotic treatment was the most common health event investigated. Ten percent of cattle were treated with antibiotics preweaning, and 16% were treated in the feedlot. Overall, 25% of cattle received therapeutic antibiotics at any time, indicating that few were treated during both the preweaning and feedlot phases.

Although general antibiotic administration was not associated with liver abscess outcomes, certain antibiotic types were. Interactions with sex and oxytetracycline (*p* = 0.01) or sex and tulathromycin (*p* ≤ 0.02) were related to liver abscess prevalence (Table 2). Therapeutic treatment with any antibiotic effective against *Fusobacterium necrophorum* (Supplementary Table S2) was not related to liver abscess outcome (results not shown).

For preweaning and lifetime data, liver abscess prevalence depended on the interaction between tulathromycin and sex (*p* ≤ 0.02), and results were consistent between time periods. Steers were more likely to have liver abscesses than heifers when neither were treated with tulathromycin (*p* < 0.01), but no sex difference was observed when both were treated with tulathromycin (*p* > 0.10). Steers treated with tulathromycin were less likely to have liver abscesses than untreated steers, especially when treatment was preweaning (*p* = 0.02). During preweaning, tulathromycin-treated steers were diagnosed with respiratory conditions, lameness, and infections at similar frequencies, while half of the heifers were diagnosed with infections, with a lower prevalence of respiratory conditions. In the feedlot, both the sexes were primarily treated with tulathromycin for respiratory conditions. This three-way interaction between antibiotics, diagnosis, and sex was not tested because of limited sample size.

In the lifetime health data, sex and oxytetracycline interacted (*p* = 0.01), although individual contrasts were not significant (Table 4). Oxytetracycline was primarily administered to treat pinkeye and foot rot with numerically different treatment patterns by sex. More heifers were treated for pinkeye, and more steers were treated for foot rot. Overall, more steers were treated with oxytetracycline across all time periods than heifers (Table 1).

### 3.3 Relationship between management and animal-level effects and liver abscesses

*p*-values for all other effects are shown in Table 3. Liver abscess prevalence differed by sex for all 3 developmental periods (*p* < 0.01). Heifers were 21% less likely than steers to have liver abscesses (odds ratio [95% CI] = 0.76 [0.63, 0.91], *p* < 0.01). Dam’s age was also significant (Supplementary Table S3). Not all dam age categories were represented in every year of data, and the results were partially confounded with each year. Slaughter age was only related to liver abscesses in the preweaning model that did not account for chlortetracycline or tylosin phosphate feeding (*p* = 0.04). Slaughter weight, heterosis, and breed type were not related to liver abscess prevalence (*p* > 0.10).

Feeding tylosin phosphate or chlortetracycline was significant. For tylosin phosphate, cattle fed during the whole finishing period were 46% less likely to have liver abscesses (odds ratio [95% CI] = 0.47 [0.24, 0.90], *p* = 0.03). However, no difference was present when cattle were fed tylosin phosphate for the 15-d transition period (odds ratio [95% CI] = 0.77 [0.42, 1.42], *p* = 0.41). The chlortetracycline feeding effect was also significant; however, the contrast comparing feeding and control within the same years was not significant (*p* = 0.96).

## 4 Discussion

Liver abscesses remain a costly and poorly understood challenge in beef production. Although extensive studies have focused on dietary strategies and prophylactic antibiotics, comparatively little is known about how therapeutic treatments and illness throughout the production cycle relate to liver abscess formation. Using a large, low-risk population with no metaphylactic antibiotic use, the present study provides new evidence that preweaning illness may have a more persistent influence on liver abscess outcomes than previously recognized.

### 4.1 Sex-specific associations between therapeutic antibiotics and liver abscesses

Broad therapeutic antibiotic use was not associated with liver abscess prevalence, aligning with prior studies showing no effect of metaphylactic antibiotics administered at feedlot arrival (9, 10). Instead, the effects were specific to antibiotic class and sex. Steers and heifers have physiological differences that may affect immunity (25) and have different liver abscess outcomes (3, 5). Greater liver abscess incidence in steers may be due to greater feed intake and more days on feed (5), although recent research found no relationship between feed intake and liver abscesses (26). Research on liver abscess sex differences has focused on quantifying differences rather than the causal mechanisms underlying them.

Tulathromycin had consistent interactions with sex in both preweaning and lifetime analyses. When cattle did not receive tulathromycin, more steers than heifers had liver abscesses, as expected from the literature (3, 5), but tulathromycin treatment masked that sex difference. Limited research has assessed tulathromycin’s effectiveness in steers and heifers, likely because including sex differences requires double the sample size. In agreement with therapeutic results, metaphylactic tulathromycin use after feedlot entry had no sex interaction related to morbidity or mortality (27, 28). Differences by production phase align with research on pharmacokinetic differences in tulathromycin between pre-ruminant and ruminant dairy calves (29), indicating that drug dosage might not scale linearly in young calves. In this case, tulathromycin could be more potent in younger calves, leading to observable differences in liver abscesses later in life.

Steers treated with tulathromycin preweaning were less likely to develop liver abscesses, a novel finding in this study. Limited research exists on beef calves treated with tulathromycin preweaning. Health data are difficult to track because of inconsistent record-keeping practices, extensive production systems, and segmentation in the beef industry. Hence, most calf research exists for dairy rather than beef calves. Therapeutic and metaphylactic tulathromycin treatment in young dairy calves had no sex differences in subsequent outcomes and did not prevent respiratory disease at weaning (30, 31). However, prophylactic tulathromycin use in dairy heifer calves was associated with greater gain and survival after first calving relative to oxytetracycline (32). These inconsistent results demonstrate the need for large, longitudinal studies including steers and heifers.

Tulathromycin’s activity against *Fusobacterium necrophorum*, the primary etiological agent of liver abscesses, makes a relationship with liver abscesses biologically plausible. Whether tulathromycin acts by reducing early *Fusobacterium* exposure, altering host susceptibility, or shifting microbial communities warrants further investigation. Existing microbiome studies have shown limited long-term effects of tulathromycin on global bacterial populations (33–37), but none have specifically targeted *Fusobacterium*, underscoring a key need for future research.

Oxytetracycline also interacts with sex in the lifetime analysis. Limited prior research showed no effect of oxytetracycline on liver abscesses but a positive effect with chlortetracycline (38). Although individual contrasts were not significant, the pattern and activity against *Fusobacterium necrophorum* suggested that sex-specific disease presentation and treatment contexts may influence abscess risk. After oxytetracycline treatment, the nasopharyngeal and fecal microbiomes were disrupted for up to 34 d (34, 39). Larger datasets—including more heifers—will be essential for clarifying these relationships.

### 4.2 Sex-specific associations between preweaning infections and liver abscesses

Heifers diagnosed with infections were less likely to have liver abscesses than heifers without infections. Preweaning infections were likely causing these results because twice as many cattle had preweaning infections as in the feedlot. The causal link between infections and fewer liver abscesses in heifers was not clear because the underlying interactions were difficult to parse given the small sample size for heifers. Given previously discussed sex interactions for tulathromycin and oxytetracycline, infection results could result from antibiotic treatments. Sex, treatment for infections, and liver abscesses could have a complex interaction. Infections are a broad category, including cellulitis, ear, eye, scrotal, and umbilicus infections, but no type was frequent enough to analyze independently and draw clear conclusions. Alternatively, an unobserved factor could be creating a non-causal correlation between infections and liver abscesses in heifers.

Contrary to expectations, neither respiratory disease nor lameness was associated with liver abscesses. Although these conditions can influence intake patterns (40, 41) and rumen function (42, 43), their relatively low prevalence in this low-risk population may have limited power to detect subtle associations. Liver abscesses have been associated with greater lung consolidation (11, 44), leading to a potential link with prior respiratory disease. In addition, lameness includes foot rot, caused by *Fusobacterium necrophorum*, leading to a possible connection with liver abscesses.

### 4.3 Management and animal-level factors related to liver abscesses

Consistent with previous research, steers had more liver abscesses than heifers (3, 5). Although liver abscesses were often associated with lighter carcass weights (3, 4, 45), live weights did not differ. Lighter carcass weights could result from additional trimming from adhered abscesses, which would not be reflected in live weight differences.

Heterosis is the change in performance of a crossbred relative to the average of its parental breeds, and no estimates of heterosis exist for liver abscesses. Heterosis is often important for disease and health traits (46) and would be expected to provide a protective effect against liver abscesses, but no effect was observed. In addition to heterosis, breed types are important considerations in breeding systems. The majority of previous breed differences focused on beef relative to dairy breeds (3, 47, 48) rather than comparisons within beef breeds. The absence of an effect of breed type or heterosis meant beef producers could continue their current breeding program without affecting liver abscess risk.

Tylosin phosphate results were consistent with the well-documented protective effect when fed during the finishing period (1, 4, 7). Chlortetracycline effects were inconsistent with prior literature showing a preventative effect (40–42), but the differences were likely due to the short feeding duration in the present study.

### 4.4 Study limitations

Although this retrospective study was relatively large compared to the liver abscess literature, the sample size was a limiting factor. The prevalence of liver abscesses was moderate relative to industry average, and few animals were treated for the illness. Hence, attempting to associate two rare events can be statistically challenging. Sample sizes were limited for interactions with sex and between health events. Designed studies are needed to verify the effect of treatment (tulathromycin and oxytetracycline) on sex interactions and the relationship with liver abscesses.

In addition, this study encompassed a single beef operation with some consistency in protocols from year to year. However, diagnosis and treatment protocols still have some subjectivity, particularly as personnel change over time. Treatment protocols change as new drugs enter the market and industry best practices shift. As changes happen, these results may become less relevant to future production practices. Additional research is needed in more diverse industry settings to confirm that these results can be replicated and are applicable to higher-risk cattle, including beef × dairy cattle, and to different antibiotic use strategies.

## 5 Conclusion

This retrospective study provides new insights into how early-life illness and antibiotic therapy relate to liver abscess development in low-risk, crossbred beef cattle. Although broad categories of diagnoses and therapeutic treatments were not consistently associated with abscess risk, two findings emerged as particularly noteworthy. First, steers treated preweaning with tulathromycin were less likely to develop liver abscesses, highlighting a potential role for early-life exposure to antibiotics active against *Fusobacterium necrophorum*, the principal pathogen involved in abscess formation. Second, heifers diagnosed with infections—primarily infectious bovine keratoconjunctivitis—had a lesser prevalence of liver abscesses, suggesting that early immune challenges or their treatment may influence later susceptibility. The biological mechanisms remain unclear but may involve differences in immune function, pathogen exposure, or microbiome dynamics that begin early in life. Importantly, the findings point toward preweaning interventions as a potentially influential—and previously underrecognized—component of liver abscess risk. Additional research is needed to validate these observational results in a designed experiment and in other production systems.

## Data Availability

The data analyzed in this study is subject to the following licenses/restrictions: the raw data supporting the conclusions of this manuscript will be made available by the authors, without undue reservation, to any qualified researcher. Requests to access these datasets should be directed to Amanda K. Lindholm-Perry, amanda.lindholm@usda.gov.

## References

[1] Broadway P Nagaraja T Lawrence T Galyean M Hales K. Liver abscesses—new perspectives on a historic fed-cattle issue. Appl Anim Sci. (2024) 40:237–43. doi: 10.15232/aas.2023-02498

[2] Taylor DD Groenendaal H Morley PS Zagmutt F. Economic costs of liver abscesses in U.S. beef feedlot cattle: a comprehensive economic analysis. J Anim Sci. (2025) 103:skaf127. doi: 10.1093/jas/skaf127, 40265468 PMC12288028

[3] Grimes B McEvers T Tennant T Johnson J Lawrence T. Relationship of liver abnormalities with carcass performance and value. Appl Anim Sci. (2024) 40:358–75. doi: 10.15232/aas.2023-02482

[4] Reinhardt C Hubbert M. Control of liver abscesses in feedlot cattle: a review. Prof Anim Sci. (2015) 31:101–8. doi: 10.15232/pas.2014-01364

[5] Nagaraja T Chengappa M. Liver abscesses in feedlot cattle: a review. J Anim Sci. (1998) 76:287–98. doi: 10.2527/1998.761287x, 9464910

[6] Champagne RE Lancaster PA White BJ Schmidt PH Mancke MR Jensen MK . Association of Liver Abscess with demographic factors, gross pathology, and gastrointestinal histologic morphology in feedyard mortalities. Transl Anim Sci. (2025) 9:txaf031. doi: 10.1093/tas/txaf031, 40191693 PMC11971717

[7] Feitoza LFBB Baker AN Thorn RL Monteiro LS Nasiu F Nagaraja TG . Targeted durations of tylosin phosphate on incidence and severity of liver abscesses in feedlot cattle. Appl Anim Sci. (2025) 41:1–9. doi: 10.15232/aas.2024-02574

[8] FDA Fact Sheet: Veterinary Feed Directive Final Rule and Next Steps. (2015) [Accessed April 04, 2025]. Available online at: https://www.fda.gov/animal-veterinary/development-approval-process/fact-sheet-veterinary-feed-directive-final-rule-and-next-steps

[9] Coppin C Smock T Helmuth C Manahan J Long N Hoffman A . The effects of administering different metaphylactic antimicrobials on growth performance and health outcomes of high-risk, newly received feedlot steers. Transl Anim Sci. (2022) 6:1–11. doi: 10.1093/tas/txac140, 36415567 PMC9673257

[10] Stegner JE Lucas MJ McLaughlin CL Davis MS Alaniz GR Weigel DJ . Comparative effects of therapeutic programs on bovine respiratory disease, performance, carcass, and profitability of high-risk feedlot heifers. Prof Anim Sci. (2013) 29:208–18. doi: 10.15232/S1080-7446(15)30226-6

[11] Grimes BB McEvers TJ Tennant TC Lawrence TE. Association of bovine lung lesions at slaughter with carcass performance, value and liver-abscess severity. Transl Anim Sci. (2025) 9:txaf112. doi: 10.1093/tas/txaf112 40909388 PMC12405687

[12] Ahlberg CM Kuehn LA Thallman RM Kachman SD Snelling WM Spangler ML. Breed effects and genetic parameter estimates for calving difficulty and birth weight in a multibreed population. J Anim Sci. (2016) 94:1857–64. doi: 10.2527/jas.2015-0161, 27285683

[13] Engle BN Thallman RM Snelling WM Wheeler TL Shackelford SD King DA . Breed-specific heterosis for growth and carcass traits in 18 U.S. cattle breeds. J Anim Sci. (2025) 103:skaf048. doi: 10.1093/jas/skaf048, 39985752 PMC11956688

[14] Bennett GL Snelling WM McDaneld TG. A "Genetics First" Approach to Selection. Proceedings World Congress on Genetics Applied in Livestock Production. Auckland, New Zealand: WCGALP (2018).

[15] Keele J Kuehn L McDaneld T Tait R Jones S Keel B . Genomewide association study of liver abscess in beef cattle. J Anim Sci. (2016) 94:490–9. doi: 10.2527/jas.2015-9887, 27065119

[16] Elanco Animal Health Liver-Check Service. (2025). [Accessed June 5, 2025]. Available online at: https://farmanimal.elanco.com/us/basic-page/services-adding-value-to-your-beef-operation

[17] R Core Team. R: A Language and Environment for Statistical Computing. Vienna, Austria: R Foundation for Statistical Computing (2024).

[18] Bates D Mächler M Bolker B Walker S. Fitting linear mixed-effects models using Lme4. J Stat Softw. (2015) 67:1–48. doi: 10.18637/jss.v067.i01

[19] Beef Improvement Federation Age of Dam: Beef Improvement Federation. (2021) (Accessed August 26, 2025]. Available online at: https://guidelines.beefimprovement.org/index.php/Age_of_Dam

[20] Akaike H. A new look at the statistical model identification. IEEE Trans Autom Control. (1974) 19:716–23. doi: 10.1109/TAC.1974.1100705

[21] Hartig F. Dharma: Residual Diagnostics for Hierarchical (Multi-Level/Mixed) Regression Models. R Package Version 0.4.7. (2024).

[22] Abdi H. "Bonferroni and Šidák corrections for multiple comparisons". In: Editor Salkind NJ. Encyclopedia of Measurement and Statistics, vol. 3 Thousand Oaks, CA: Sage (2007). p. 2007.

[23] Lenth R. Emmeans: Estimated Marginal Means, Aka Least-Squares Means. R Package Version 1.11.2. (2025).

[24] Tukey JW. Comparing individual means in the analysis of variance. Biometrics. (1949) 5:99–114. doi: 10.2307/3001913, 18151955

[25] Klein SL. The effects of hormones on sex differences in infection: from genes to behavior. Neurosci Biobehav Rev. (2000) 24:627–38. doi: 10.1016/S0149-7634(00)00027-0, 10940438

[26] Bradford HL Kuehn LA Keel BN Neville BW Lindholm-Perry AK. Seasonal relationship between liver abscesses and feeding behavior in crossbred beef cattle. Transl Anim Sci. (2026) 10:txag053. doi: 10.1093/tas/txag053 42205589 PMC13202452

[27] McAtee TB Booker CW Hunsaker BD McMullen CA Fenton RK Raaphorst HS . Comparing initial antimicrobial treatments for bovine respiratory disease in feedlot cattle following tulathromycin metaphylaxis. Bovine Pract. (2025) 60:22–32. doi: 10.21423/bpj20269280

[28] Burciaga-Robles LO McMullen C Bras A Guerra-Maupome M Nava-Gaspar RA Rademacher RD . An evaluation of arrival metaphylaxis with enrofloxacin compared to tulathromycin in feedlot cattle at high risk of developing bovine respiratory disease in Mexico. Bov Pract. (2022) 56:53–9. doi: 10.21423/bovine-vol56no1p53-59

[29] Mzyk DA Bublitz CM Hobgood GD Martinez MN Smith GW Baynes RE. Effect of age on the pharmacokinetics and distribution of tulathromycin in interstitial and pulmonary epithelial lining fluid in healthy calves. Am J Vet Res. (2018) 79:1193–203. doi: 10.2460/ajvr.79.11.1193, 30372149

[30] Binversie ES Ruegg PL Combs DK Ollivett TL. Randomized clinical trial to assess the effect of antibiotic therapy on health and growth of Preweaned dairy calves diagnosed with respiratory disease using respiratory scoring and lung ultrasound. J Dairy Sci. (2020) 103:11723–35. doi: 10.3168/jds.2019-18044, 33222860

[31] Madureira Ferreira M Santos B Skarbek A Mills C Thom H Prentice D . Bovine respiratory disease (Brd) in post-weaning calves with different prevention strategies and the impact on performance and health status. Animals. (2024) 14:2807. doi: 10.3390/ani14192807, 39409755 PMC11476203

[32] Stanton AL Kelton DF LeBlanc SJ Wormuth J Leslie KE. The effect of respiratory disease and a preventative antibiotic treatment on growth, survival, age at first calving, and milk production of dairy heifers. J Dairy Sci. (2012) 95:4950–60. doi: 10.3168/jds.2011-5067 22916899

[33] Johnson BT White BJ Amachawadi RG Kleinhenz MD Farney JK Shippy TD . Determining the effect of varied proportions of cohort administered tulathromycin at arrival on nasopharyngeal microbiota and performance characteristics in yearling steers in the first 56 days on feed. Microorganisms. (2024) 12:2512. doi: 10.3390/microorganisms12122512, 39770715 PMC11677586

[34] Holman DB Yang W Alexander TW. Antibiotic treatment in feedlot cattle: a longitudinal study of the effect of oxytetracycline and tulathromycin on the Fecal and nasopharyngeal microbiota. Microbiome. (2019) 7:86. doi: 10.1186/s40168-019-0696-4, 31167657 PMC6549328

[35] Foditsch C Pereira RVV Siler JD Altier C Warnick LD. Effects of treatment with enrofloxacin or tulathromycin on Fecal microbiota composition and genetic function of dairy calves. PLoS One. (2019) 14:e0219635. doi: 10.1371/journal.pone.0219635, 31825967 PMC6905572

[36] Martin CC Baccili CC Avila-Campos MJ Hurley DJ Gomes V. Effect of prophylactic use of tulathromycin on gut bacterial populations, inflammatory profile and Diarrhea in newborn Holstein calves. Res Vet Sci. (2021) 136:268–76. doi: 10.1016/j.rvsc.2021.02.026, 33721714

[37] Doster E Rovira P Noyes NR Burgess BA Yang X Weinroth MD . Investigating effects of tulathromycin Metaphylaxis on the Fecal Resistome and microbiome of commercial feedlot cattle early in the feeding period. Front Microbiol. (2018) 9:1715. doi: 10.3389/fmicb.2018.01715, 30105011 PMC6077226

[38] Theurer ME Amachawadi RG. Antimicrobial and biological methods to control liver abscesses. Vet Clin North Am Food Anim Pract. (2022) 38:383–94. doi: 10.1016/j.cvfa.2022.07.001, 36243460

[39] Rovira P. Short-term impact of oxytetracycline administration on the Fecal microbiome, Resistome and virulome of grazing cattle. Antibiotics. (2023) 12:470. doi: 10.3390/antibiotics12030470, 36978337 PMC10044027

[40] Wolfger B Schwartzkopf-Genswein KS Barkema HW Pajor EA Levy M Orsel K. Feeding behavior as an early predictor of bovine respiratory disease in north American feedlot systems. J Anim Sci. (2015) 93:377–85. doi: 10.2527/jas.2013-8030, 25568380

[41] González LA Tolkamp BJ Coffey MP Ferret A Kyriazakis I. Changes in feeding behavior as possible indicators for the automatic monitoring of health disorders in dairy cows. J Dairy Sci. (2008) 91:1017–28. doi: 10.3168/jds.2007-0530, 18292258

[42] Lean IJ Westwood CT Golder HM Vermunt JJ. Impact of nutrition on lameness and claw health in cattle. Livest Sci. (2013) 156:71–87. doi: 10.1016/j.livsci.2013.06.006, 38826717

[43] Owens FN Secrist DS Hill WJ Gill DR. Acidosis in cattle: a review. J Anim Sci. (1998) 76:275–86. doi: 10.2527/1998.761275x, 9464909

[44] Herrick RT Rogers CL Jones TA McEvers TJ Brown TR Maxwell CL . Association of liver-abscess presence and severity with trim loss, slaughter yield, carcass grading performance, lung lesions, and value of fed-Holsteins. Appl Anim Sci. (2024) 40:376–85. doi: 10.15232/aas.2023-02471

[45] Hales K. Summary of the special issue on liver abscesses in cattle and thoughts on future research. Appl Anim Sci. (2024) 40:430–6. doi: 10.15232/aas.2024-02553

[46] Bunning H Wall E Chagunda MGG Banos G Simm G. Heterosis in cattle crossbreeding schemes in tropical regions: Meta-analysis of effects of breed combination, trait type, and climate on level of heterosis. J Anim Sci. (2019) 97:29–34. doi: 10.1093/jas/sky406, 30346552 PMC6313114

[47] Herrick R Rogers C McEvers T Amachawadi R Nagaraja T Maxwell C . Exploratory observational quantification of liver abscess incidence, specific to region and cattle type, and their associations to viscera value and bacterial flora. Appl Anim Sci. (2022) 38:170–82. doi: 10.15232/aas.2021-02228

[48] Grote A Fanning T DeVuyst EA Grigsby Z Crosswhite J Beck P. A commercial scale evaluation of the effects of post-weaning management system of dairy × beef hybrid steers compared to native beef steers on performance, carcass characteristics, and net returns. Transl Anim Sci. (2025) 9:txaf118. doi: 10.1093/tas/txaf118 40980501 PMC12448398

